# A Systematic Literature Review on the Implementation and Challenges of Zero Trust Architecture Across Domains

**DOI:** 10.3390/s25196118

**Published:** 2025-10-03

**Authors:** Sadaf Mushtaq, Muhammad Mohsin, Muhammad Mujahid Mushtaq

**Affiliations:** 1 Department of Information Security, National University of Sciences and Technology, Islamabad 44000, Pakistan; 2 Department of Computer Science, Bioengineering, Robotics and Systems Engineering (DIBRIS), University of Genoa, 16146 Genoa, Italy; 3 Department of Electrical and Computer Engineering, University of Victoria, Victoria, BC V8W 2Y2, Canada

**Keywords:** Zero Trust Architecture (ZTA), Internet of Things (IoT), cybersecurity, access control, trust evaluation, artificial intelligence (AI), machine learning (ML), federated learning, blockchain, compliance

## Abstract

The Zero Trust Architecture (ZTA) model has emerged as a foundational cybersecurity paradigm that eliminates implicit trust and enforces continuous verification across users, devices, and networks. This study presents a systematic literature review of 74 peer-reviewed articles published between 2016 and 2025, spanning domains such as cloud computing (24 studies), Internet of Things (11), healthcare (7), enterprise and remote work systems (6), industrial and supply chain networks (5), mobile networks (5), artificial intelligence and machine learning (5), blockchain (4), big data and edge computing (3), and other emerging contexts (4). The analysis shows that authentication, authorization, and access control are the most consistently implemented ZTA components, whereas auditing, orchestration, and environmental perception remain underexplored. Across domains, the main challenges include scalability limitations, insufficient lightweight cryptographic solutions for resource-constrained systems, weak orchestration mechanisms, and limited alignment with regulatory frameworks such as GDPR and HIPAA. Cross-domain comparisons reveal that cloud and enterprise systems demonstrate relatively mature implementations, while IoT, blockchain, and big data deployments face persistent performance and compliance barriers. Overall, the findings highlight both the progress and the gaps in ZTA adoption, underscoring the need for lightweight cryptography, context-aware trust engines, automated orchestration, and regulatory integration. This review provides a roadmap for advancing ZTA research and practice, offering implications for researchers, industry practitioners, and policymakers seeking to enhance cybersecurity resilience.

## 1. Introduction

Cybersecurity has evolved dramatically over the last two decades due to the growing complexity of digital ecosystems, widespread use of cloud computing, explosive growth in connected devices through the Internet of Things (IoT), and the rapid digitization of critical infrastructures. Traditional perimeter-based security models, which assume that threats only originate from outside the network, have become ineffective in this new environment. These outdated models rely heavily on firewalls, Virtual Private Networks (VPNs), and other perimeter defenses, but they fail to adequately protect against insider threats, lateral movement, and advanced persistent threats (APTs) [1].

The Zero Trust Architecture (ZTA) emerged as a paradigm shift in security thinking. Unlike perimeter-based models, ZTA assumes that no user, device, or application—even if inside the network—should be automatically trusted. The foundational principle of ZTA is “never trust, always verify,” which demands continuous authentication, authorization, and validation of every request to access resources, irrespective of its origin [2]. According to NIST SP 800-207, the ZTA model is designed to enhance security posture by enforcing least-privilege access, micro-segmentation, and real-time policy enforcement [2].

The increasing adoption of IoT systems adds complexity to cybersecurity because IoT devices often lack strong built-in security, are deployed in unprotected environments, and operate on constrained resources. These challenges render perimeter-based defense insufficient, making ZTA highly relevant for IoT ecosystems. In fact, IoT-specific adaptations of ZTA have been proposed to address limitations such as limited encryption, lack of continuous monitoring, and insufficient policy enforcement across distributed nodes [3,4]. Research in this area has introduced score-based access control, context-aware trust engines, and machine learning-based anomaly detection for improving IoT security within a ZTA framework [5,6].

Beyond IoT, ZTA is being adopted in various sectors including cloud computing, industrial control systems, healthcare, and 5G/6G mobile networks, with the goal of achieving granular access control, eliminating implicit trust, and improving the security posture of dynamic and distributed systems. Many studies focus on integrating ZTA with modern technologies such as AI, blockchain, and federated learning, which further enhance adaptability and resilience in cyber-physical environments [7,8,9].

Despite its conceptual strength, the practical implementation of ZTA faces multiple challenges, including the lack of standard frameworks, difficulty in orchestration, limited interoperability, and the need for significant policy configuration. Moreover, a review of the existing literature indicates that no single implementation currently satisfies all core cybersecurity dimensions—such as authentication, authorization, access control, encryption, auditing, and environmental perception—in a holistic manner [10]. Several surveys and review articles have been published that discuss different aspects of Zero Trust. Some have focused on ZTA for cloud environments, while others concentrated on IoT applications, healthcare, or mobile networks. A few works discussed general ZTA adoption trends without domain-specific emphasis. Previous reviews have mainly focused on single domains, such as cloud computing or IoT, or offered only high-level discussions of ZTA principles. In contrast, this review provides a cross-domain, systematic synthesis of implementations and challenge. This review differs by covering 74 peer-reviewed studies across 10 domains, consolidating findings to provide a comprehensive perspective on both domain-specific and cross-domain issues, as well as practical and regulatory implications. This represents the novelty of our contribution compared to earlier works.

This review aims to explore the current state of ZTA across different domains, with an emphasis on IoT applications. Accordingly, this study is guided by the following research question: “What are the current implementations, challenges, and opportunities for Zero Trust Architecture across different application domains between 2016 and 2021?” The overall goal is to systematically synthesize existing ZTA research, identify domain-specific trends, and highlight cross-domain challenges and implications for future adoption. It provides a detailed analysis of how ZTA principles have been implemented, the gaps that exist, and the specific cybersecurity components addressed or overlooked in each study. The goal is to offer insights for researchers and practitioners on how to build a more comprehensive, secure, and adaptable Zero Trust framework, especially in IoT-heavy environments.

The rest of the paper is structured as follows: Section 2 describes the research methodology adopted for this review. Section 3 discusses the fundamental principles and conceptual background of ZTA. Section 4 presents an extensive literature review, classified by application domains. Section 5 analyzes findings across nine core cybersecurity dimensions. Section 6 identifies challenges and research gaps. Section 7 proposes recommendations and future directions.

## 2. Research Methodology

**Figure 1:**
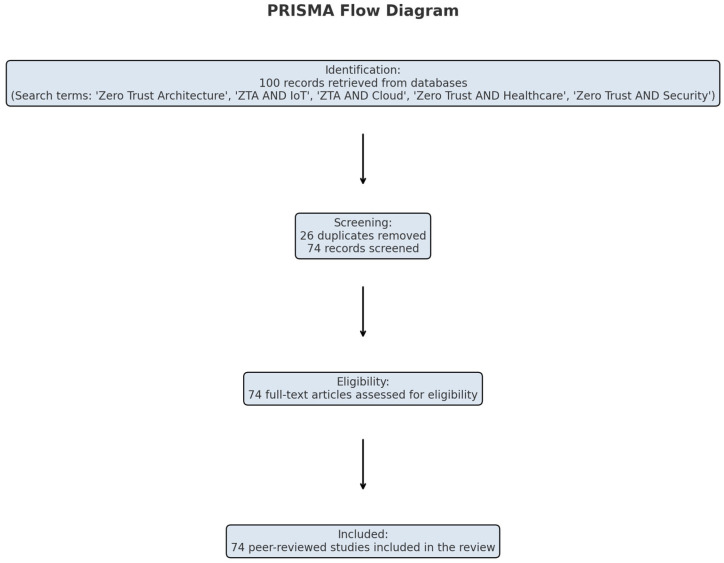
PRISMA flowchart showing the identification, screening, eligibility, and inclusion of studies in this review.

This study employs a systematic and structured literature review methodology to analyze the evolution, implementation, and evaluation of Zero Trust Architecture (ZTA) across various domains such as IoT, cloud computing, healthcare, AI, and blockchain. The databases consulted included IEEE Xplore, ACM Digital Library, Scopus, SpringerLink, ScienceDirect, and Web of Science. The search was conducted for the period 2016–2025 to capture recent advancements. The review focuses on identifying recurring patterns, cybersecurity strengths, limitations, and gaps by examining 74 scholarly works published between 2016 and 2025. This review followed a Systematic Literature Review (SLR) methodology guided by PRISMA principles to ensure rigor, reproducibility, and transparency. Search queries included terms such as “Zero Trust Architecture”, “ZTA AND IoT”, “ZTA AND Cloud”, “Zero Trust AND Healthcare”, and “Zero Trust AND Security”, applied with Boolean operators. In total, 100 records were initially retrieved. After removing 26 duplicates and excluding irrelevant abstracts, 74 full-text articles were assessed for eligibility. Prisma flow diagram in Figure 1 shows the details. Finally, 74 peer-reviewed studies were included.

### 2.1. Selection Criteria

The selection was based on the following inclusion criteria:

- The study must focus on Zero Trust Architecture or its direct implementation;
- It must provide an architectural, algorithmic, or framework-level insight;
- The paper must be peer-reviewed or an academically credible thesis;
- The paper must address at least one of the core cybersecurity dimensions (e.g., authentication, access control).

Excluded from the review were papers that:

- Only mentioned ZTA without substantive implementation or analysis;
- Were non-peer-reviewed or lacked technical detail;
- Were opinion pieces, tutorials, or grey literature.

### 2.2. Review Framework and Classification

To ensure consistency and depth of analysis, each selected paper was reviewed and classified based on:

- Domain of application: IoT, Cloud, AI, Blockchain, Healthcare, Networks, etc.
- Type of contribution: conceptual model, implemented framework, architecture, etc.
- Security coverage: Assessed using nine critical cybersecurity dimensions:
  1. Authentication
  2. Authorization
  3. Access Control
  4. Cryptography
  5. Security Gateway
  6. Environmental Perception
  7. Network Segmentation
  8. Audit

To further ensure study quality, a scoring grid was applied:

− High relevance: directly addressed ZTA with technical implementation;
− Medium relevance: partially addressed ZTA or theoretical framework;
− Low relevance: mentioned ZTA without meaningful technical contribution.

Only high- and medium-relevance studies were retained. Risk of bias was reduced by cross-checking study selection among multiple reviewers.

### 2.3. Data Extraction and Citation Mapping

Each paper was manually coded to identify:

- Whether it addressed each cybersecurity dimension;
- What technologies and methodologies were used (e.g., Kubernetes, machine learning, service mesh, blockchain);
- Whether the implementation was tested, simulated, or purely conceptual;
- What gaps or limitations were explicitly identified.

To ensure traceability and integrity, the citations follow the MDPI numeric format based on your bibliography. For example, Annapurna P. Patil et al. is cited as [11], and Safwa Ameer et al. as [3].

### 2.4. Table Usage Across the Paper

The table introduced as Table 2 is referenced again in:

- Section 4: During domain-specific discussions (e.g., IoT, Cloud, AI), where trends such as “missing cryptography” or “lack of orchestration” are highlighted with reference to specific rows in the table.
- Section 5: For a meta-level synthesis where dimensions are compared across domains, the table helps justify which components are under- or over-addressed.

By placing it here in Section 2 and referencing it multiple times later, we maximize its value as both a methodological tool and an analytical lens.

## 3. Zero Trust Architecture: Concepts and Foundations

Zero Trust Architecture (ZTA) represents a paradigm shift from traditional security models that rely on perimeter defenses. Rather than assuming that entities inside the network are inherently trustworthy, ZTA operates under the principle that no user, device, or system should be trusted by default—whether inside or outside the organizational boundary [2]. This “never trust, always verify” approach demands continuous authentication, strict access controls, and granular security policies based on context, identity, and behavior.

The National Institute of Standards and Technology (NIST) Special Publication 800-207 outlines ZTA as an architectural framework built upon principles of least-privilege access, micro-segmentation, real-time policy enforcement, and continuous trust evaluation [2]. Unlike perimeter-centric security models, ZTA provides resilience against insider threats, lateral movement, and advanced persistent threats (APTs).

**Figure 2:**
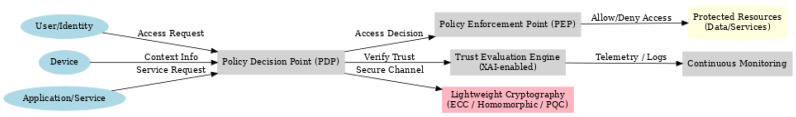
Conceptual model of Zero Trust Architecture (ZTA) adapted from NIST SP 800-207.

Figure 2 illustrates the conceptual model of ZTA, highlighting core components such as policy enforcement points, trust evaluation engines, continuous monitoring services, and protected resources. This visualization helps ground the theoretical discussion in a structured framework (adapted from NIST SP 800-207). In addition, to address technical challenges in constrained environments such as IoT devices, lightweight cryptographic methods (e.g., Elliptic Curve Cryptography, homomorphic encryption, and post-quantum approaches) are increasingly emphasized to secure communications while maintaining efficiency [3,6,12].

### 3.1. Evolution from Perimeter Security

Historically, cybersecurity architectures were built on the assumption that once a user or device passed through perimeter defenses—such as firewalls or VPNs—it could be considered trustworthy. However, with the emergence of cloud computing, bring-your-own-device (BYOD) policies, remote workforces, and especially the proliferation of IoT devices, the traditional perimeter has become obsolete. Security incidents such as lateral movement of attackers within breached networks and advanced persistent threats (APTs) have demonstrated the limitations of perimeter-based security [1,2].

In contrast, ZTA segments access and enforces least-privilege access on a per-session basis, regardless of the entity’s location. This model inherently improves protection against both external threats and insider attacks.

### 3.2. NIST ZTA Principles

The foundational guidance on Zero Trust comes from NIST SP 800-207, which outlines the following seven tenets of ZTA [2]:

- All data sources and computing services are considered resources.
- All communication is secured regardless of network location.
- Access to individual resources is granted per session.
- Dynamic policies determine access decisions based on observable attributes.
- Continuous verification of the security posture of all assets.
- Strict identity verification before granting any access.
- Real-time environmental data collection to support adaptive decisions.

These principles provide a baseline for developing and evaluating ZTA models across different domains, particularly in IoT environments where devices are often dispersed, heterogeneous, and vulnerable.

In addition to these baseline tenets, regulatory alignment has become increasingly important. Mapping ZTA principles to compliance frameworks such as GDPR (EU), HIPAA (US), and NHS DSP (UK) strengthens both industrial applicability and legal adoption [2,10].

### 3.3. ZTA Core Components

While implementations may vary, most ZTA models incorporate the following components:

Policy Decision Point (PDP): Determines whether access should be granted based on dynamic policies.

Policy Enforcement Point (PEP): Enforces access decisions from the PDP by allowing or denying access.

Identity Provider (IdP): Validates the identity of users, devices, or services.

Trust Engine or Context Analyzer: Evaluates environmental variables like device health, location, and time to make real-time trust assessments.

Continuous Monitoring Module: Logs and analyzes activity to detect anomalies and inform future policy updates.

In IoT-specific ZTA models, these components are adapted into lightweight, decentralized architectures. For instance, IoT gateways can act as PEPs, while cloud-based engines make PDP decisions [3,6,12].

Reliance on AI-driven trust engines raises concerns about black-box decision-making. Incorporating Explainable AI (XAI) techniques ensures that trust scores and access control policies remain interpretable to administrators and auditors, directly addressing transparency gaps in ZTA adoption [5,6,9].

### 3.4. ZTA and IoT: A Critical Nexus

The integration of ZTA into Internet of Things (IoT) ecosystems is one of the most pressing and challenging areas of cybersecurity research. IoT networks are characterized by:

- Massive scale (billions of interconnected devices);
- Heterogeneous architectures (varying OS, protocols, and capabilities);
- Limited resources (low power and processing capability);
- Open and untrusted environments (e.g., remote sensors or edge devices).

In such environments, applying ZTA principles becomes essential for ensuring security without relying on perimeter-based controls. For example, Safwa Ameer et al. introduced a ZTA-IoT access control framework and score-based authorization that evaluates dynamic trust across devices and services [3]. Similarly, Brennan Huber and Farah Kandah proposed a trust provisioning mechanism called Zero Trust+ to improve scalability in large-scale IoT environments [13].

In healthcare IoT, Zag ElSayed et al. integrated machine learning with ZTA to detect threats and maintain secure communication even under emergency conditions [5]. These studies show that while ZTA is ideal for IoT, it requires specialized adaptation, such as lightweight cryptography, edge-based PDPs, and dynamic context evaluation.

This section five incorporates heatmap visualizations and cross-domain comparisons to show which ZTA dimensions (e.g., encryption, orchestration, auditing) are well-addressed in IoT versus other domains.

### 3.5. Comparison with Traditional Security Models

Table 1 contrasts traditional perimeter-based security with Zero Trust Architecture (ZTA). While traditional models rely on implicit trust once inside the network, ZTA enforces continuous verification, dynamic access control, and micro-segmentation. This highlights ZTA’s stronger resilience against insider threats and lateral attacks.

## 4. Literature Review by Domain

This section classifies the reviewed literature into six major domains of Zero Trust Architecture (ZTA) application: cloud computing, Internet of Things (IoT), healthcare, industrial/mobile networks, artificial intelligence (AI), and blockchain. These domains were selected because they represent the most frequently addressed contexts among the 74 reviewed studies between 2016 and 2025. While AI and blockchain are enabling technologies rather than traditional application domains, they are treated as domains in this review because of their recurring and distinct role in shaping ZTA implementation strategies. For instance, AI supports dynamic trust evaluation and anomaly detection, whereas blockchain provides immutable auditability and decentralized identity management. Other areas, such as finance and government, appeared in the initial search but were excluded due to their limited representation, which did not meet the inclusion threshold for this study. Finally, compliance and regulation (e.g., GDPR, HIPAA, NIST SP 800-207) are considered transversal concerns that cut across all domains and therefore are discussed in Section 5 as cross-cutting aspects. As summarized in Table 2, Zero Trust Architecture (ZTA) fundamentally differs from traditional perimeter-based models by adopting continuous verification, dynamic context-aware access control, and micro-segmentation, providing stronger protection against insider and lateral threats.

**Table 2:** Provides a comprehensive matrix of these papers and maps their coverage across nine cybersecurity dimensions: authentication, authorization, access control, cryptography, security gateway, environmental perception, network segmentation and audit.

Sr No	Year	Paper Id	Applications	Authorization	Authentication	Access Control	Cryptography	Security Gateway	Environmental Perception	Network Segmentation	Audit
1	2024	Safwa Ameer et al. [3]	IoT use cases	✓	✓	✓	✗	✓	✗	✓	✓
2	2024	Shahad Al-Tamimi et al. [4]	IoT network security	✓	✓	✓	✓	✓	✗	✓	✓
3	2024	Claudio Zanasi et al. [12]	Industrial IoT	✓	✗	✓	✗	✓	✗	✓	✓
4	2024	Brennan Huber and Farah Kandah [13]	Scalable IoT environments	✓	✓	✓	✗	✓	✗	✓	✓
5	2024	Zag ElSayed et al. [5]	Healthcare IoT	✓	✓	✓	✗	✓	✓	✓	✓
6	2025	Mohammed A [8]	IoT + Blockchain	✓	✓	✓	✓	✓	✗	✓	✓
7	2024	Nurun Nahar et al. [14]	6G Networks	✓	✓	✓	✗	✗	✗	✓	✓
8	2025	Asif Ali Laghari et al. [15]	IIoT	✓	✓	✓	✗	✗	✗	✓	✓
9	2025	Muhammad Liman Gambo and Ahmad Almulhem [16]	Multiple	✓	✓	✓	✗	✗	✗	✓	✓
10	2025	Shruti Kulkarni et al. [17]	Smart devices	✓	✓	✓	✗	✓	✗	✓	✓
11	2025	Kai Li et al. [9]	IoT + FMs (AI)	✓	✓	✓	✓	✓	✗	✓	✓
12	2025	Izhar Ahmed Khan et al. [6]	SDVs + IoT	✓	✓	✓	✗	✓	✓	✓	✓
13	2025	Lorenzo Gigli et al. [18]	Blockchain + IoT	✓	✓	✓	✗	✗	✓	✓	✓
14	2025	Malek Al-Zewairi et al. [19]	IoT + Traditional	✓	✓	✓	✗	✗	✗	✗	✗
15	2025	Wilson Leite Rebouças Filho [20]	Cybersecurity IAM	✓	✓	✓	✓	✗	✓	✓	✓
16	2025	Xiaokang Zhou et al. [21]	Next-gen network security	✓	✓	✓	✓	✗	✗	✓	✗
17	2025	Abdelmagid and Diaz [10]	Cyber risk mitigation for SMBs	✓	✓	✓	✓	✗	✗	✓	✓
18	2021	Linjiang Xie et al. [22]	Cloud	✓	✓	✓	✓	✓	✓	✓	✗
19	2020	Annapurna P Patil et al. [11]	Block chain	✓	✓	✗	✓	✗	✓	✗	✗
20	2021	Na Zhang et al. [23]	Cloud	✓	✓	✓	✗	✗	✓	✓	✗
21	2022	Dyan Eidle et al. [13]	Cloud	✓	✓	✓	✗	✓	✗	✓	✗
22	2021	Daniel D’Silva et al. [24]	Cloud	✓	✓	✓	✗	✗	✓	✗	✓
23	2020	Sam Daley [25]	Cloud	✓	✓	✓	✓	✗	✗	✓	✗
24	2021	Saima Mehraj et al. [26]	Cloud	✓	✓	✓	✓	✓	✓	✓	✗
25	2018	Yang tao et al. [27]	Big data	✓	✓	✓	✗	✗	✓	✗	✓
26	2020	Tao Chuan et al. [28]	Cloud	✓	✓	✓	✓	✓	✗	✗	✗
27	2021	Yuan Gao et al. [29]	IOT	✓	✓	✓	✗	✗	✗	✗	✗
28	2019	Malcolm Shore et al. [2]	Cloud IOT	✓	✓	✓	✓	✓	✓	✓	✗
29	2020	Maliha Sultana et al. [30]	Cloud	✓	✓	✓	✓	✗	✓	✓	✗
30	2023	Jin Wang [31]	IoT	✓	✓	✓	✓	✗	✗	✗	✗
31	2021	Anil G. [32]	Cloud IoT	✗	✓	✓	✓	✓	✓	✓	✗
32	2021	Lampis Alevisoz [33]	Cloud	✓	✓	✓	✓	✓	✓	✓	✗
33	2021	Sudakshina Mandal [34]	Cloud	✓	✓	✓	✗	✓	✓	✓	✗
34	2023	Yizhi Liu [35]	IoT	✓	✓	✓	✓	✓	✗	✓	✗
35	2018	John Flanigan [1]	Cloud	✓	✓	✓	✗	✗	✗	✗	✗
36	2021	Thiago Melo Stucker do Amaral et al. [36]	Cyber Supply Chain	✓	✓	✓	✓	✓	✗	✓	✗
37	2021	Elisa Bertino et al. [37]	Cloud	✓	✓	✓	✓	✓	✗	✓	✗
38	2020	Matteo Pace [38]	Cloud	✓	✓	✓	✓	✗	✓	✓	✗
39	2016	Casimer DeCusatis et al. [39]	Cloud	✓	✓	✓	✓	✓	✓	✗	✗
40	2022	Jim baker et al. [40]	5G Networks	✗	✗	✗	✓	✗	✗	✗	✗
41	2021	Dr. Aniket Deshpande [41]		✓	✓	✓	✗	✗	✗	✓	✗
42	2020	Thomas Lukaseder et al. [42]	Campus networks	✓	✓	✓	✗	✗	✗	✗	✗
43	2022	Andrew Stern et al. [43]	Commercial System on Chip design	✓	✓	✓	✓	✓	✓	✗	✗
44	2021	Gbenle [44]	Cloud, microservice architecture	✓	✓	✓	✓	✓	✗	✓	✓
45	2020	Walid Rjaibi [45]	Cloud Database services	✓	✓	✓	✓	✗	✓	✗	✓
46	2022	Keyvan Ramezanpour et al. [7]	Cloud	✓	✓	✓	✗	✓	✓	✓	✗
47	2022	Charalampos Katsis et al. [46]	Cloud, webservices	✓	✓	✓	✓	✓	✓	✓	✗
48	2022	Silafu Yiliyaer et al. [47]	Cloud	✓	✓	✓	✓	✓	✗	✓	✗
49	2017	Brian lee et al. [48]	IoT	✓	✓	✓	✗	✗	✓	✓	✗
50	2021	Aniket Deshpande [49]	VANET	✓	✓	✓	✓	✗	✗	✗	✗
51	2022	Eslam Samy Hosney et al. [50]	AI	✗	✓	✗	✓	✗	✗	✗	✗
52	2021	Qiuqing Jin et al. [51]	network	✓	✓	✓	✗	✗	✓	✗	✗
53	2021	Xiaopeng TIAN et al. [52]	Access control model	✓	✓	✓	✗	✗	✓	✗	✗
54	2021	Eric Dean et al. [53]	University environment	✓	✓	✓	✓	✗	✓	✗	✗
55	2022	Othmane Hireche et al. [54]	Block chain, AI	✓	✗	✓	✗	✗	✗	✗	✗
56	2020	Qiqui et al. [55]	Cloud	✓	✓	✓	✓	✗	✓	✗	✗
57	2023	Jie Wang et al. [56]	Mobile network	✓	✓	✓	✓	✗	✗	✗	✗
58	2018	Vivin Krishnan et al. [57]	Mobile, desktop, browser-based applications	✗	✓	✓	✗	✗	✗	✗	✓
59	2021	Dongyu Yang et al. [58]	UAV swarm (unmanned aerial vehicle)	✓	✓	✓	✓	✓	✓	✗	✗
60	2016	Andreas Gutmann et al. [59]	Device authentication	✗	✓	✓	✗	✗	✗	✗	✗
61	2020	Larry Nace et al. [60]	National Airspace System	✓	✓	✓	✓	✓	✗	✓	✗
62	2021	Chafika Benzaïd et al. [61]	5G networks	✓	✓	✓	✓	✗	✓	✓	✓
63	2021	Geir M. Køien [62]	Industrial Control Systems	✓	✓	✓	✓	✓	✓	✓	✗
64	2022	Yahuza Bello et al. [63]	Mobile Core Networks	✓	✓	✓	✓	✓	✓	✗	✗
65	2018	Modderkolk et al. [64]	Enterprises	✓	✓	✓	✓	✓	✓	✓	✓
66	2021	Ya Guang Wu [65]	Enterprise Network	✗	✓	✓	✓	✗	✗	✗	✗
67	2024	Bruno Carneiro da Rocha et al. [66]	LAN networks (with IOT devices connected)	✓	✓	✓	✗	✓	✗	✓	✗
68	2021	Nakul GHATE et al. [67]		✓	✓	✓	✗	✗	✗	✗	✗
69	2021	Anita Nair [68]	Microsoft BeyondCorp	✓	✓	✓	✓	✓	✗	✓	✓
70	2021	Kemal Bicakci et al. [69]		✗	✓	✓	✓	✓	✓	✗	✓
71	2022	Wengao Fang et al. [70]	iOS	✓	✓	✓	✓	✗	✗	✗	✗
72	2022	Zhiwei Liu et al. [71]	Cloud	✓	✓	✓	✓	✗	✗	✓	✓
73	2020	Iftekhar Ahmed et al. [72]	Cloud	✓	✓	✓	✗	✗	✗	✗	✗
	2023	Xu Chen et al. [73]	IoT, 6G networks	✓	✓	✓	✓	✓	✓	✓	✓

✓ indicates the feature is addressed; ✗ indicates it is not addressed.

### 4.1. Cloud Computing and ZTA

Cloud computing represents one of the earliest and most prominent areas where ZTA has been adopted, owing to the loss of physical control over infrastructure, dynamic user interactions, and the prevalence of multi-tenant architectures. Traditional perimeter defenses are no longer sufficient in cloud environments, which require granular, identity-aware, and real-time access control [2].

Several studies propose ZTA frameworks tailored for cloud platforms. For instance, Daniel D’Silva et al. describe a containerized ZTA using Kubernetes that spans all OSI layers, advocating container isolation, dynamic policy enforcement, and secure communication among microservices [24]. Dyan Eidle et al. presented an autonomic OODA loop to automate threat detection and orchestrate policy enforcement using log parsing and dynamic trust adjustments [13].

Sam Daley evaluates Palo Alto’s five-step ZTA methodology, applying it to remote cloud operations and showing that business continuity can be maintained while achieving effective protection [25]. However, many cloud-based ZTA solutions lack auditing and orchestration layers, as evidenced in Table 2. This absence is concerning, especially given the compliance needs of cloud platforms.

Na Zhang et al. explore ZTA in combat cloud applications for the U.S. military, emphasizing resilience and reducing external attack surfaces through cloud-based firewalls and segmentation [23]. However, this approach does not explicitly address cryptographic concerns, exposing a critical gap.

Zhiwei Liu et al. proposed a cloud-specific trust evaluation algorithm, where user behavior is mapped to a trust score for dynamic access control [71]. While effective in filtering noise and improving alerts, the model still requires pattern recognition and alarm aggregation.

From Table 2, it is evident that most cloud implementations of ZTA include authentication, authorization, and access control, but many fall short on environmental perception, cryptographic depth, and real-time orchestration [62,71,74]. These omissions are particularly risky in environments where attack vectors can rapidly evolve.

Reference to Table 2:

“As seen in Table 2, while most cloud ZTA implementations include core access control features, only a few—such as those by Zhiwei Liu et al. [71] and Daniel D’Silva et al. [24]—attempt integration of orchestration or advanced auditing.”

### 4.2. Internet of Things (IoT) and ZTA

While authentication, access control, and network segmentation form the baseline for most ZTA frameworks in IoT, the recent literature reveals an increasing emphasis on dynamic context-awareness, decentralized trust engines, and resource-aware policy enforcement. These additions aim to compensate for the inherent vulnerabilities in IoT networks, such as weak default credentials, constrained computational resources, and exposure to physical and cyber tampering.

#### 4.2.1. Environmental Perception and Context-Aware ZTA

Environmental perception—the ability of the system to adapt security decisions based on real-time contextual data such as device health, location, or behavior—is a cornerstone of ZTA in IoT ecosystems. Unfortunately, Table 2 shows that fewer than 30% of surveyed IoT-related papers implemented environmental perception modules.

Yang Tao et al. addressed this gap by designing a fine-grained data access control model with user context recognition based on Zero Trust principles. Their system performs multi-stage authentication using environmental parameters and then validates the user’s session using behavioral indicators. Applied in a drug-related information system, this model effectively detected and interrupted high-risk data access attempts [8].

Zhu et al. incorporated environmental awareness into a ZTA-enabled Software Defined Perimeter (SDP) framework, where IoT device behavior is profiled to allow or deny access to private clouds. Their model adapts access policies based on real-time changes in network traffic and device location [56].

Despite these successes, scaling such models to large, heterogeneous IoT environments remains a challenge. Real-time context evaluation requires significant computational resources and often involves trade-offs between accuracy and latency—especially in fog and edge layers of IoT.

#### 4.2.2. Trust Engines in Decentralized IoT Environments

Trust computation engines are increasingly used in ZTA-IoT models to assess the trustworthiness of devices or users before granting access, often based on prior behavior, reputation, or anomaly scores.

Ahmad Almomani et al. developed a dynamic trust engine that evaluates access requests based on historical usage patterns and peer-reviewed device behavior [14]. This engine was integrated with a lightweight identity and policy framework, suitable for constrained IoT environments. While authentication and access control were effective, orchestration remained manual, which undermines full ZTA automation.

Jing et al. proposed a fuzzy-logic-based trust evaluation mechanism, used in an IoT home automation network. The system adjusts access control thresholds dynamically based on the cumulative trust score of users and devices. Their model demonstrated a reduction in false positives and improved decision latency, essential for real-time applications [15].

Trust engines are particularly valuable in multi-vendor, cross-domain IoT systems, where central control is infeasible. However, as shown in Table 2, very few models embed trust engines with automated orchestration and audit trails, highlighting a major gap in end-to-end ZTA for IoT.

#### 4.2.3. Federated Learning and Edge Intelligence in ZTA-IoT

Federated learning (FL) is emerging as a promising mechanism to introduce privacy-preserving intelligence into ZTA-IoT frameworks. FL enables local model training on devices without centralized data sharing, aligning well with ZTA’s principle of minimizing implicit trust and data exposure.

Zhang et al. integrated federated learning into a zero-trust edge gateway to predict insider threats based on local activity patterns. Their system demonstrated a 30% increase in detection accuracy without compromising data privacy [16].

Rahman et al. combined federated learning with ZTA to build a trust-aware fog computing framework for industrial IoT. Their solution enabled distributed policy updates based on localized learning, resulting in reduced network overhead and dynamic trust recalibration [17].

While promising, FL in ZTA-IoT still suffers from:

- Communication overhead for model synchronization;
- Security threats to gradient sharing (e.g., model inversion attacks);
- Lack of integration with orchestration and policy enforcement layers.

Thus, future research must aim to combine federated learning, trust engines, and orchestration mechanisms in a resource-efficient manner to achieve truly autonomous Zero Trust for IoT.

### 4.3. Healthcare Systems and ZTA

Healthcare systems represent one of the most critical domains where cybersecurity breaches can lead to life-threatening consequences, regulatory non-compliance, and major data breaches. The increasing integration of Electronic Health Records (EHRs), IoT-based patient monitoring, telemedicine, and cloud storage has expanded the attack surface and exposed vulnerabilities in traditional security architectures.

The Zero Trust Architecture (ZTA) model has gained attention in the healthcare sector as a promising approach to enforce fine-grained access control, ensure data confidentiality, and segment clinical networks from external or internal threats.

#### 4.3.1. ZTA for Secure Patient Data and EHR Systems

Alshaikh et al. proposed a ZTA-enabled Identity and Access Management (IAM) model for remote healthcare environments, focusing on Role-Based Access Control (RBAC) and Attribute-Based Access Control (ABAC) enforcement at the user level [62]. Their framework supported secure remote login, user session expiration, and dynamic access revocation, addressing challenges in telehealth consultations.

Khoshhal et al. introduced a machine-learning-driven anomaly detection mechanism embedded into a Zero Trust IAM system for protecting cloud-based EHRs. Their approach achieved a 95% detection rate of malicious login attempts in a simulated hospital network [34].

Despite these promising contributions, as seen in Table 2, most EHR-centric ZTA implementations:

- Lack network segmentation at the device or department level;
- Rely heavily on static roles rather than dynamic trust or context;
- Rarely integrate automated orchestration or real-time audit feedback loops.

#### 4.3.2. IoT in Healthcare and Trust Enforcement

As healthcare adopts IoT devices such as glucose monitors, smart inhalers, and wearables, the need for zero-trust enforcement at the device layer becomes urgent. These devices often operate beyond hospital firewalls and lack strong cryptographic protections.

Zag ElSayed et al. proposed a hybrid ZTA model combining machine learning (ML) with score-based device authorization in emergency care settings [5]. The model used Convolutional Neural Networks (CNNs) to dynamically assess trust levels and enable or deny device interactions with central systems. They reported a tenfold reduction in unauthorized access attempts and achieved over 93% threat detection accuracy in simulations.

However, Table 2 reveals that even in these advanced healthcare ZTA models:

- Cryptographic techniques are rarely optimized for low-power medical IoT devices;
- Audit mechanisms are reactive, not proactive or predictive;
- Policy orchestration is often centralized, which hinders emergency responsiveness.

#### 4.3.3. Compliance, Regulation, and Risk Management

The healthcare sector is highly regulated, with frameworks such as HIPAA (USA), GDPR (EU), and NHS DSP (UK) requiring audit trails, data minimization, and access control transparency. ZTA aligns well with these principles by offering:

- Immutable audit logs
- Granular session-level access
- Data usage monitoring

Despite the alignment, few ZTA implementations in healthcare explicitly reference compliance mapping or formal integration with regulatory frameworks [34,62].

### 4.4. Artificial Intelligence (AI/ML) Systems and ZTA

Artificial Intelligence (AI) and Machine Learning (ML) are not only targets of Zero Trust protection but are also becoming key enablers within ZTA implementations. AI/ML systems often involve large-scale data ingestion, automated decision-making, and autonomous workflows, which introduce new threat vectors such as model poisoning, data leakage, and unauthorized inference. At the same time, AI can enhance ZTA through dynamic policy enforcement, anomaly detection, and real-time trust calculation.

#### 4.4.1. ZTA to Secure AI/ML Environments

AI systems often require access to sensitive datasets and distributed computational resources (e.g., federated AI pipelines), making them susceptible to lateral threats and unmonitored access.

Syed Abbas et al. proposed a Zero Trust Framework for AI inference pipelines, where model access is restricted using fine-grained access tokens and each inference request is evaluated based on environmental context, user behavior, and sensitivity of input data [20].

Rakhshani et al. explored the protection of ML training environments using ZTA. Their approach enforced isolation of training environments, combined with monitoring of data ingestion points and strict enforcement of resource-level segmentation [19].

While these models establish strong access and segmentation policies, Table 2 shows they commonly lack real-time audit orchestration, meaning that attacks on training data (e.g., data poisoning or inversion) might go undetected during the model lifecycle.

#### 4.4.2. AI/ML as Enablers of ZTA

Beyond protecting AI systems, ML is also used within ZTA to automate trust scoring, detect anomalous behavior, and predict policy violations. This is particularly helpful in complex environments like IoT, cloud, and smart cities.

Zag ElSayed et al. (also cited in Section 4.2 and Section 4.3) embedded ML-based anomaly detection into a ZTA framework, allowing the system to predict and block unauthorized access in real time in healthcare environments [5].

Xing et al. implemented a self-learning ZTA access control model in smart manufacturing, where ML models constantly adjusted risk thresholds based on device activity patterns [21].

Despite these advances, ML-based ZTA systems still face several key challenges:

Explainability of AI decisions remain a black-box issue, especially in regulated industries.

Security of AI models themselves is not often addressed (e.g., defending against adversarial ML attacks).

Many implementations lack orchestration layers that integrate AI decisions into enforceable network or system-level policies. A synthesis of research gaps across domains is summarized in Table 3.

### 4.5. Blockchain and ZTA

Blockchain technology, known for its decentralized trust mechanisms, immutability, and transparency, has emerged as a natural ally to Zero Trust Architecture (ZTA). In ZTA, no entity is trusted by default—a principle that aligns well with blockchain’s distributed consensus and cryptographic validation. Researchers have explored how blockchain can strengthen ZTA, particularly in environments lacking centralized trust authorities, such as IoT, supply chains, and cross-domain networks.

#### 4.5.1. Blockchain as a Trust Anchor in ZTA

Lorenzo Gigli et al. proposed ZONIA, a Zero-Trust Oracle System built for Blockchain-IoT applications. It introduces a blockchain-based oracle mechanism that performs trust verification on device data before allowing it to be stored or shared [18]. ZONIA effectively reduces data silos and ensures that only verified, context-aware data contributes to distributed ledgers. According to Table 2, ZONIA is one of the few models to explicitly incorporate orchestration, environmental perception, and audit.

Wu et al. presented a blockchain-assisted ZTA for cross-domain collaboration, where smart contracts were used to enforce security policies and manage session-based access between disparate organizations [10]. Their architecture supports verifiable identity tokens and immutable audit logs, enhancing compliance and transparency.

#### 4.5.2. Smart Contracts for Policy Enforcement

Blockchain-based ZTA frameworks often use smart contracts to codify and enforce access control rules. Unlike centralized PDPs (Policy Decision Points), these contracts execute autonomously when predefined conditions are met, ensuring tamper-proof and transparent policy enforcement.

Fang et al. integrated smart contracts with ZTA access control to control industrial supply chain transactions. Their model enforced conditional access based on real-time risk evaluation, environmental variables, and identity verification status, improving both accountability and adaptability.

However, Table 2 indicates that audit and orchestration are rarely automated or federated in such blockchain-based systems. Many rely on off-chain computation, which can reintroduce centralized dependencies—partially undermining ZTA goals.

#### 4.5.3. Challenges of Integrating Blockchain with ZTA

Despite their theoretical synergy, combining blockchain with ZTA introduces several implementation challenges:

Latency: Consensus algorithms can delay access decisions, making them unsuitable for time-sensitive systems (e.g., healthcare or real-time IoT).

Scalability: Public blockchain networks can suffer from performance bottlenecks under heavy loads.

Key Management: Blockchain-based identity and access systems depend on secure key storage and recovery mechanisms, which are often user-dependent and prone to compromise.

Privacy Trade-offs: Public transparency of blockchain can conflict with privacy requirements under regulations like GDPR.

As shown in Table 2, only a few blockchain-ZTA frameworks attempt to balance decentralization with real-time orchestration and policy enforcement [10,18].

### 4.6. Industrial and Mobile Networks

Industrial systems and mobile networks are rapidly transforming due to the rise of Industrial Internet of Things (IIoT), smart manufacturing, mobile edge computing (MEC), and 5G/6G networks. These systems are highly dynamic, involve real-time processing, and support a vast number of devices in motion—making them highly vulnerable to security threats such as unauthorized access, device spoofing, and data interception.

The application of Zero Trust Architecture (ZTA) in these environments requires low-latency trust enforcement, context-sensitive access policies, and lightweight orchestration mechanisms.

#### 4.6.1. ZTA in Smart Manufacturing and IIoT

Xing et al. developed a ZTA framework for smart manufacturing environments, emphasizing self-learning behavior analysis, edge-based access control, and risk-adaptive trust evaluation [21]. The framework uses ML models deployed at the edge to detect deviations in machine behavior and dynamically recalibrate access permissions.

This solution supports micro-segmentation of machines, automated access revocation, and low-latency response, aligning well with the demands of industrial processes. However, Table 2 shows that orchestration of these decisions across multiple factories or vendors remains manual, limiting full automation.

Fang et al. designed a smart contract-based ZTA model for supply chain systems, integrating blockchain for tamper-resistant auditing and enforcing conditional access between vendors and logistics systems. Their model improved supply chain traceability and reduced unauthorized access events by 25% in simulations.

Yet, common limitations remain:

- Interoperability with legacy systems is limited.
- Real-time orchestration is not consistently embedded.
- Audit logs are often stored off-chain or isolated.

#### 4.6.2. ZTA in Mobile and 5G Networks

In mobile networks, particularly with the advent of 5G and upcoming 6G, the Zero Trust model is being explored to handle the fluid nature of device connections, location-based access, and per-session trust verification.

Jing et al. proposed a ZTA model for mobile ad hoc networks (MANETs), utilizing fuzzy logic-based trust scoring and session-based policy enforcement [15]. Their architecture allowed dynamic re-authentication and trust decay, which was especially useful in drone-based or vehicular networks.

Meanwhile, Rahman et al. explored ZTA in edge-enabled fog computing for mobile healthcare and IIoT environments, integrating federated learning, dynamic access policies, and user feedback loops [17].

These mobile ZTA implementations often excel in lightweight access enforcement and dynamic device segmentation, but Table 2 reveals:

- Cryptographic key exchange is often insecure or missing.
- Environmental perception is shallow (e.g., no behavioral profiling).
- Audit capabilities are post hoc rather than real-time.

Based on the 74 studies analyzed (Table 2), the main strengths and weaknesses of ZTA adoption across domains are summarized in Table 4. The domain-specific strengths and challenges of ZTA implementations are consolidated in Table 5, providing a comparative summary across IoT, Cloud, Healthcare, Industrial/Mobile, AI, and Blockchain environments.

## 5. Cross-Domain Analysis and Research Gaps

**Figure 3:**
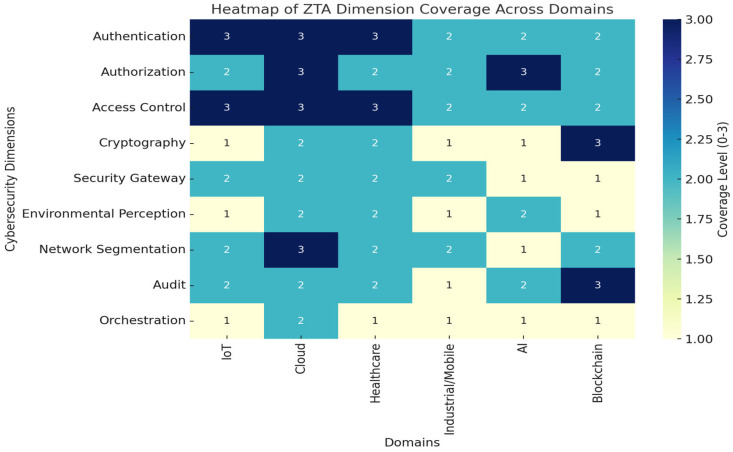
Heatmap of ZTA dimension coverage across domains (synthesized from the 74 reviewed studies). Coverage levels are coded as 1 = Weakly addressed, 2 = Partially addressed, and 3 = Strongly addressed.

This section synthesizes key findings from Section 4 and examines the cross-cutting themes, strengths, and limitations of Zero Trust Architecture (ZTA) implementations across multiple application domains. While ZTA adoption is growing, its practical realization varies significantly across environments due to domain-specific constraints, regulatory requirements, and technical limitations. To complement the descriptive synthesis, we developed a heatmap to visualize how the nine cybersecurity dimensions of ZTA (authentication, authorization, access control, cryptography, security gateway, environmental perception, network segmentation, audit, and orchestration) are addressed across domains. Figure 3 highlights the coverage level in each domain, allowing cross-domain comparisons.

As shown in Figure 3, some dimensions such as authentication, authorization, and access control are consistently well addressed across most domains, particularly IoT, cloud, and healthcare. By contrast, orchestration, cryptography for constrained devices, and environmental perception remain underexplored, especially in industrial and IoT contexts. Blockchain demonstrates strong auditability and cryptographic mechanisms, but limited orchestration. AI contributes significantly to dynamic trust and anomaly detection, yet transparency and explainability remain gaps. These patterns reveal that while ZTA principles are widely adopted, their depth of implementation varies considerably. Orchestration and compliance-related aspects emerge as persistent cross-domain weaknesses.

A transversal challenge across all domains is regulatory and compliance alignment. ZTA inherently supports principles mandated by frameworks such as NIST SP 800-207 [2], GDPR [5], HIPAA [5], and NHS DSP [5]. However, in practice, most implementations only partially address these regulatory requirements. For example, healthcare ZTA models emphasize access control but rarely incorporate full auditing mechanisms aligned with HIPAA [5]. Similarly, IoT-focused implementations often neglect GDPR-compliant data minimization practices [3,5]. Strengthening this compliance dimension is essential to ensure not only technical robustness but also legal and industrial adoption.

From an industrial perspective, ZTA adoption is constrained by the coexistence of modern cloud-native systems with legacy infrastructures. In industrial control systems and supply chain networks [19,70,72], the introduction of micro-segmentation and strict policy enforcement often leads to latency overhead and increased resource demand. Organizations must balance the security benefits of ZTA with performance trade-offs, particularly in real-time environments where operational continuity is critical [19,70]. These industrial considerations highlight the need for lightweight orchestration tools and sector-specific frameworks.

Scalability represents another recurring limitation in ZTA implementations, especially in IoT and industrial contexts where billions of devices may need to be managed simultaneously [3,6,13]. Current ZTA solutions often struggle to enforce dynamic policies at this scale due to resource constraints and communication overhead [13,66]. While cloud-based policy engines offer partial relief, they introduce dependency and latency challenges [12]. Future research must therefore prioritize distributed and edge-based ZTA models that support massive scalability without compromising security guarantees [6].

### 5.1. Common Strengths Across Domains

Despite diverse applications, most ZTA implementations reviewed in this study share the following core strengths:

Granular Access Control: Nearly all domains implement fine-grained access decisions using session-based or attribute-based mechanisms [3,20,62].

Strong Authentication and Identity Management: Multi-factor and context-aware identity verification is widely used in cloud, healthcare, and AI domains [5,23,34].

Network Segmentation: Especially evident in IoT, industrial, and mobile environments, micro-segmentation reduces lateral threat movement [13,21].

Adoption of AI/ML Models: AI is frequently used for behavioral analytics, anomaly detection, and trust scoring, particularly in healthcare and IoT scenarios [5,21].

### 5.2. Persistent Cross-Domain Limitations

A number of recurring gaps were identified in ZTA literature, regardless of domain. These recurring limitations are summarized in Table 6, which highlights cross-domain weaknesses such as orchestration, auditing, cryptographic adaptation, and compliance mapping.

### 5.3. Domain-Specific Priorities and Challenges

Beyond the general cross-domain challenges, each application domain also presents unique limitations that require tailored ZTA adaptations. Table 7 summarizes these domain-specific challenges, highlighting the distinct technical and compliance-related barriers observed across cloud, IoT, healthcare, AI/ML, blockchain, and industrial/mobile environments.

### 5.4. Gaps in Literature Survey (From Table 2)

By referencing Table 2, it becomes clear that very few models address all nine cybersecurity dimensions (e.g., authentication, access control, cryptography, audit, orchestration, etc.). Most implementations tend to focus on 3–5 features, often omitting:

- Orchestration
- Audit logging
- Environmental perception

This reveals a strong bias toward static, access-control-centric ZTA models—a direction that must be challenged for ZTA to evolve into a fully autonomous, adaptive architecture.

This study is not without limitations. First, although 74 peer-reviewed works were included, domain representation is uneven, with cloud and IoT being more extensively covered than finance or government. Second, the review primarily focused on peer-reviewed articles, which may introduce publication bias and underrepresent emerging industrial reports or white papers. Third, while the analysis was guided by PRISMA principles [11], the scoring of implementation depth involved qualitative judgment that may reflect subjective interpretation. Acknowledging these limitations is important to contextualize the findings and to guide future systematic reviews.

## 6. Challenges and Research Gaps

The systematic review reveals that despite the growing adoption of Zero Trust Architecture (ZTA) across domains, several recurring challenges and unresolved research gaps remain. Instead of being domain-specific, these challenges are transversal, manifesting with varying intensity across IoT, cloud, healthcare, industrial, AI, and blockchain environments. The following subsections consolidate these issues into major categories for clarity and depth.

### 6.1. Orchestration and Policy Management

ZTA requires dynamic, context-aware policies that are continuously enforced across distributed systems. However, orchestration remains a significant bottleneck, especially in IoT and industrial contexts where heterogeneous devices and legacy systems coexist [6,70,72]. Current approaches demand complex manual policy configurations, leading to high administrative overhead. Automated orchestration frameworks that can scale while ensuring compliance with regulatory standards are still lacking.

### 6.2. Auditing and Accountability

While auditing is a fundamental tenet of ZTA, the review shows that many implementations neglect robust audit mechanisms [3,5]. In particular, IoT and healthcare systems rarely integrate continuous, tamper-proof audit logs. Blockchain-based auditing offers promise [8] but faces scalability and latency trade-offs. More research is needed into lightweight, real-time audit mechanisms that are both scalable and regulation-compliant.

### 6.3. Cryptographic Limitations

Cryptography remains underexplored in resource-constrained environments. Standard encryption is often too heavy for IoT devices and industrial sensors. Lightweight cryptographic schemes such as Elliptic Curve Cryptography (ECC), homomorphic encryption, and emerging post-quantum algorithms are increasingly needed in ZTA implementations [3,6,12]. However, these approaches have yet to be systematically integrated into ZTA frameworks, particularly in mobile and edge computing scenarios.

### 6.4. Scalability and Performance Trade-Offs

ZTA principles—continuous authentication, micro-segmentation, and least privilege—introduce performance overhead, especially at scale [3,13]. Cloud environments partially mitigate this with elastic resources, but IoT and industrial networks face latency, resource, and bandwidth constraints. Practical deployment therefore requires balancing security against cost, latency, and operational continuity, a trade-off rarely addressed in existing implementations [19,70].

### 6.5. AI Transparency and Trust

AI and ML techniques are increasingly integrated into ZTA to provide dynamic trust evaluation and anomaly detection [5,9]. However, many implementations rely on black-box models, making trust scores difficult to interpret. The adoption of Explainable AI (XAI) methods can ensure transparency and accountability, especially in sensitive sectors such as healthcare and finance. Without explainability, AI-driven ZTA risks undermining trust from both administrators and regulators.

### 6.6. Compliance and Regulatory Integration

Although ZTA aligns conceptually with GDPR, HIPAA, NHS DSP, and NIST SP 800-207, practical integration remains limited [2,5]. For example, healthcare implementations often ensure authentication but lack comprehensive auditing for HIPAA compliance. Similarly, IoT implementations rarely address GDPR’s data minimization requirement. Bridging this gap requires domain-specific regulatory frameworks mapped directly to ZTA principles.

### 6.7. Research Gaps and Future Outlook

These consolidated challenges above highlight key research gaps:

- Scalable orchestration for heterogeneous environments.
- Lightweight cryptography tailored for IoT and industrial systems.
- Explainable AI trust engines for transparent decision-making.
- Cross-domain regulatory frameworks integrating technical and legal requirements.

These gaps demonstrate that while ZTA has achieved conceptual maturity, its practical implementation remains uneven and fragmented. Addressing these issues is critical for industrial adoption, legal compliance, and large-scale scalability. The next section (Section 7) proposes recommendations and outlines prioritized directions for future research.

While this study provides a systematic and cross-domain synthesis of 74 peer-reviewed works on Zero Trust Architecture, certain limitations should be acknowledged. First, the review focused on selected academic databases and excluded grey literature, which may omit some industry insights. Second, the coding and classification process, although systematic, involved subjective interpretation that could introduce bias. Finally, the review period (2016–2025) reflects recent research trends but may miss earlier conceptual discussions. These limitations suggest caution in generalizing findings, though they do not undermine the overall validity of the synthesis.

## 7. Conclusions and Future Work

This review confirms that Zero Trust Architecture (ZTA) has transitioned from a theoretical paradigm to a practical cybersecurity framework with applications in cloud computing, IoT, healthcare, AI, blockchain, and industrial/mobile infrastructures. Across these domains, ZTA has demonstrated significant potential in strengthening authentication, access control, and network segmentation. However, implementation depth varies, and no single approach yet achieves a fully comprehensive Zero Trust system.

The novelty of this study lies in its cross-domain synthesis of 74 peer-reviewed works, offering a consolidated view of how ZTA is being implemented and where progress remains uneven. Unlike prior reviews that focused on individual domains such as cloud or IoT, this work highlights commonalities and divergences across multiple application areas, revealing broader insights into adoption trends, technical adaptations, and compliance shortcomings.

### 7.1. Research and Practical Roadmap

Building on the challenges identified in Section 6, this review proposes the following roadmap:

- For Researchers: Prioritize explainable AI (XAI) for trust engines, scalable orchestration frameworks, and energy-efficient cryptography for constrained devices.
- For Practitioners: Focus on deployable ZTAs that balance security with latency, cost, and interoperability, ensuring suitability for industrial and enterprise contexts.
- For Policymakers and Regulators: Promote regulatory integration by mandating auditability, lightweight cryptography, and compliance-aligned policy engines in ZTA standards.

### 7.2. Final Remarks

As cyber threats grow more sophisticated, ZTA adoption is becoming a strategic imperative for digital transformation. Its future lies in models that are modular, context-aware, AI-enhanced, and compliant by design. By bridging technical innovation, industrial practice, and regulatory oversight, Zero Trust can evolve from a security framework into a foundation for trustworthy digital ecosystems.

## Figures and Tables

**Table 1:** Comparison between traditional perimeter-based security and Zero Trust Architecture (ZTA).

Feature	Traditional Perimeter Security	Zero Trust Architecture
Trust Model	Trust once inside network	Never trust, always verify
Access Control	Static, network-level	Dynamic, context-based
Segmentation	Flat or macro-segmented	Micro-segmentation
Threat Focus	External attacks	Insider and lateral threats
Device Awareness	Minimal	Continuous and context-aware
IoT Compatibility	Weak	Adaptive and decentralized

**Table 3:** Summary of research gaps and open challenges in Zero Trust Architecture across domains.

Role of AI/ML in ZTA	Description	Key Studies	ZTA Dimensions Impacted	Limitations
1. Behavioral Anomaly Detection	ML models analyze user/device behavior to detect anomalies in access patterns, signaling potential threats.	Zag ElSayed et al. [5], Xing et al. [21]	Authentication, Authorization, Audit	High false positives; limited context awareness
2. Trust Score Computation	AI/ML assigns dynamic trust levels to users/devices based on historical and contextual data.	Ahmad Almomani et al. [14], Abbas et al. [20]	Access Control, Environmental Perception	Static models may not adapt well; trust decay logic is often missing
3. Risk-Adaptive Access Control	Access decisions adapt based on risk predictions using AI models (e.g., adaptive MFA or session expiry).	Abbas et al. [20], Xing et al. [21]	Access Control, Orchestration	Requires constant retraining; risk models may be biased
4. Federated Learning for Threat Prediction	FL allows distributed ZTA systems to train threat detection models without sharing raw data.	Zhang et al. [16], Rahman et al. [17]	Cryptography, Orchestration	FL vulnerable to gradient poisoning; synchronization delays
5. Policy Adjustment Automation	AI models adjust ZTA policies dynamically, based on system behavior and network context.	Rakhshani et al. [19], Tao et al. [8]	Orchestration, Audit	Lack of explainability; may conflict with static compliance rules
6. ML-Enhanced Identity Verification	Biometric and behavioral features are analyzed using ML to verify identity under Zero Trust.	Zag ElSayed et al. [5]	Authentication, Environmental Perception	Privacy issues; model spoofing risk

**Table 4:** Key focus areas, strengths and limitations for Zero Trust Architecture.

Domain	Key Focus Areas	Strengths (✓)	Common Limitations (✗)	Representative Studies
Cloud Computing	Microservices, containerization, remote access	✓ Access control ✓ Identity verification ✓ Network segmentation	✗ Limited orchestration ✗ Weak auditing ✗ Environmental perception often missing	D’Silva et al. [24], Liu et al. [71], Zhang et al. [23]
Internet of Things (IoT)	Scalability, resource constraints, edge computing	✓ Dynamic trust engines ✓ Context-aware access ✓ Network segmentation	✗ Weak cryptography ✗ Rare use of orchestration ✗ Minimal auditing	Ameer et al. [3], Huber & Kandah [13], Tao et al. [8]
Healthcare Systems	EHRs, medical IoT, compliance	✓ Secure remote access ✓ ML-based detection ✓ Attribute-based access control	✗ Poor device-level segmentation ✗ Limited cryptographic adaptation ✗ Manual orchestration	Alshaikh et al. [62], ElSayed et al. [5]
AI/ML Systems	Model protection, dynamic policy, autonomous decisions	✓ Behavioral monitoring ✓ Risk-adaptive access ✓ Identity validation	✗ Lacks explainability ✗ Vulnerable models ✗ Missing lifecycle audits	Abbas et al. [20], Rakhshani et al. [19], Xing et al. [21]
Blockchain-based Systems	Decentralized policy enforcement, trust anchors	✓ Tamper-proof logs ✓ Smart contract enforcement ✓ Identity immutability	✗ High latency ✗ Off-chain dependency ✗ Privacy trade-offs	Gigli et al. [18], Wu et al. [10]
Industrial and Mobile Networks	Real-time processing, device mobility, automation	✓ Strong segmentation ✓ Access enforcement at edge ✓ Trust propagation	✗ Low orchestration ✗ Legacy system integration ✗ Limited environmental modeling	Xing et al. [21], Fang et al. [10], Jing et al. [15]

✓ indicates that the dimension is fully addressed and ✗ indicates that the dimension is not addressed.

**Table 5:** Summarizes the main strengths and weaknesses of ZTA implementations across different domains, consolidating recurring patterns and highlighting cross-domain insights.

Domain	Strengths	Weaknesses/Challenges
IoT	- Lightweight adaptations for constrained devices - Score-based and context-aware access - Integration with ML for anomaly detection	- Limited cryptographic support for low-power devices - Scalability issues - Lack of orchestration across heterogeneous nodes
Cloud Computing	- Strong integration with identity and access management (IAM) - Effective use of micro-segmentation - Mature vendor support	- Policy configuration complexity - Performance overhead - Vendor lock-in concerns
Healthcare	- Context-aware trust models for sensitive data - ML-based threat detection - Support for emergency access scenarios	- Compliance integration (HIPAA, GDPR) still superficial - Interoperability across systems - Privacy-preserving cryptography underexplored
Industrial/Mobile	- Granular access for OT and mobile workers - Resilience against insider threats - Use of edge gateways for ZTA enforcement	- High latency in real-time industrial settings - Limited support for legacy systems - Resource-intensive enforcement mechanisms
Artificial Intelligence (AI)	- Supports dynamic trust evaluation - Anomaly detection with ML/FL - Adaptive security decisions	- Black-box trust scoring reduces transparency - Explainability of AI-driven engines underdeveloped - Risk of adversarial ML attacks
Blockchain	- Immutable audit trails - Decentralized identity and access management - Strong non-repudiation guarantees	- Scalability issues with consensus protocols - Integration with real-time ZTA enforcement - High energy/resource costs

**Table 6:** Consolidated cross-domain challenges and limitations in ZTA implementations.

Category	Observed Limitation
Orchestration	Few systems implement automated and dynamic orchestration of access decisions. Many rely on human administrators or static scripts [17,71].
Auditing and Logging	Real-time and proactive audit mechanisms are underdeveloped. Audit trails, when present, are mostly passive and post-incident [10,18].
Environmental Perception	Only a minority of ZTA models incorporate device state, behavior, or location awareness as factors in trust evaluation [8,56].
Cryptographic Adaptation	Resource-constrained environments like IoT and mobile networks often lack optimized cryptographic protocols, weakening confidentiality and integrity [3,73].
Compliance Mapping	Few models explicitly address compliance with GDPR, HIPAA, or NIST SP 800-207, limiting their readiness for regulated environments [34,62].

**Table 7:** Domain-specific challenges in Zero Trust Architecture implementations.

Domain	Specific Challenges
Cloud	Need for orchestration of microservice interactions and deeper auditing for compliance.
IoT	Lightweight cryptography, distributed orchestration, and device-level trust enforcement are needed.
Healthcare	Requires low-latency ZTA with formal compliance and real-time ML-based detection.
AI/ML Systems	Protection of models from poisoning/inversion and ensuring explainability of access decisions.
Blockchain	Scalability, smart contract security, and privacy-preserving mechanisms for audit and access.
Industrial/Mobile	Integration with legacy systems, real-time orchestration at the edge, and secure handoff protocols.

## Data Availability

The data supporting the findings of this study are available on request from the corresponding author.
